# First Trimester Diagnosis of Holoprosencephaly Secondary to a Ring Chromosome 7

**DOI:** 10.1155/2013/578202

**Published:** 2013-02-21

**Authors:** Lindsay B. Henderson, Virginia L. Corson, Daniel O. Saul, Cynthia Anderson, Sarah Millard, Denise A. S. Batista, Karin J. Blakemore, Cheryl DeScipio

**Affiliations:** ^1^Institute of Genetic Medicine, Johns Hopkins University School of Medicine, Baltimore, MD 21287, USA; ^2^Department of Gynecology and Obstetrics, Johns Hopkins University School of Medicine, Baltimore, MD 21287, USA; ^3^Department of Pathology, Johns Hopkins University School of Medicine, 600 North Wolfe Street, Park Building SB202, Baltimore, MD 21287, USA; ^4^Cytogenetics Laboratory, Kennedy Krieger Institute, Baltimore, MD 21287, USA

## Abstract

Holoprosencephaly (HPE) is a developmental defect in humans in which the forebrain fails to completely separate into two hemispheres. We describe a 12 3/7-week-old fetus found on ultrasound evaluation to have features consistent with HPE, including a single anterior ventricle, fused thalami, and a flattened profile. Cytogenetic analysis of chorionic villi revealed a ring chromosome 7 [r(7)]. This uncommon finding has been associated with growth delay, microcephaly, and dermatologic abnormalities. However, both the clinical features and the extent of cytogenetic imbalance of chromosome 7 are variable, and few reported cases of r(7) have been molecularly studied. To our knowledge, this is the first report of a prenatally identified r(7), molecularly characterized using array comparative genomic hybridization.

## 1. Introduction

Ring chromosome 7 [r(7)] is a rare cytogenetic abnormality, with only 17 postnatal cases reported. The most common clinical findings associated with r(7) are growth delay, microcephaly, and dermatologic abnormalities; however, a spectrum of other features has also been described, including two cases with holoprosencephaly (HPE), a failure of the forebrain to completely separate into two hemispheres during development. Phenotypic severity of r(7) is likely correlated with the extent of cytogenetic imbalance of chromosome 7, although few reported cases have been molecularly studied to characterize the gene content lost or gained by the ring chromosome, if any. Here, we present the first report of a prenatally identified r(7), molecularly characterized using array comparative genomic hybridization.

## 2. Case Presentation

**Figure 1:**
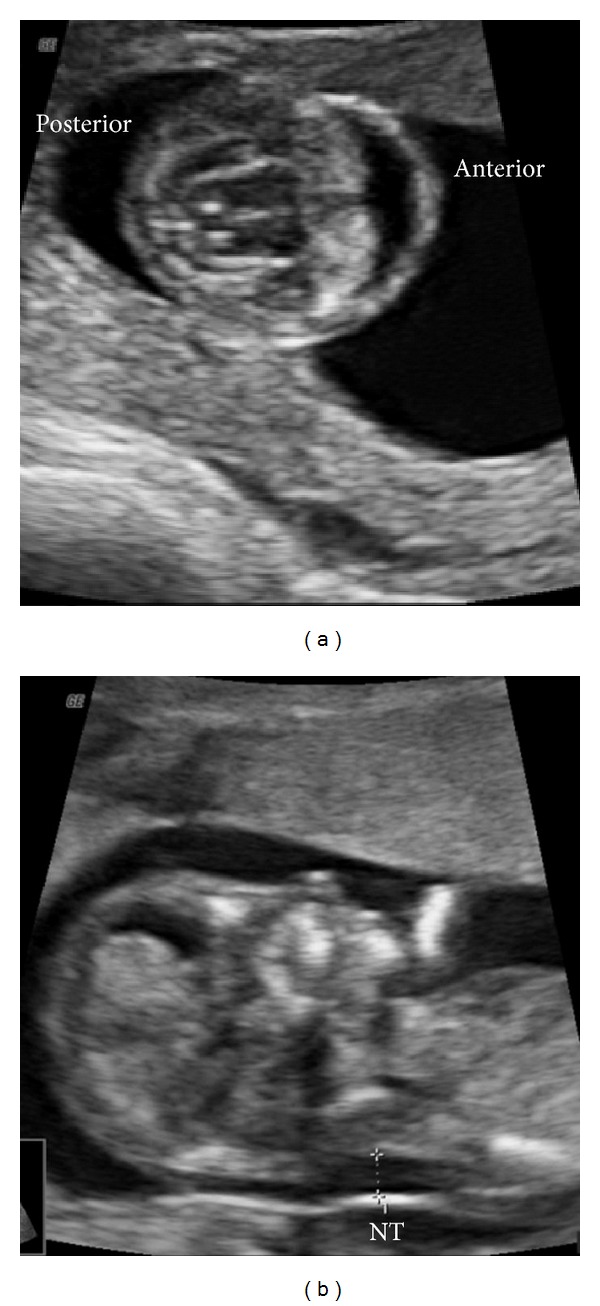
Ultrasound images at 12 3/7 weeks gestation. (a) Transverse view of the fetal intracranial anatomy demonstrates a single anterior ventricle and fused thalami; (b) sagittal view of the fetal profile demonstrates an increased nuchal translucency (NT) of 2.9 mm and a flattened profile with absent nasal bone.

A 31-year-old gravida 2 para 1001 Caucasian woman presented for first trimester screening at 12 weeks 3 days gestation from last menstrual period. Her husband was a 29-year-old Caucasian male who had a niece with Down syndrome. The couple had one healthy child and the family history was otherwise unremarkable. Ultrasound evaluation revealed a single anterior ventricle, fused thalami, an increased nuchal translucency measuring 2.9 mm, a flattened profile with an absent nasal bone, and skin edema of the thorax and abdomen (Figure 1). These findings were most consistent with a form of HPE, undifferentiated due to the early gestational age. Possible etiologies were discussed, and the patient elected to proceed with chorionic villus sampling (CVS). A subsequent ultrasound evaluation at 14 weeks 3 days confirmed the previous ultrasound examination findings and also revealed hypotelorism.

**Figure 2:**
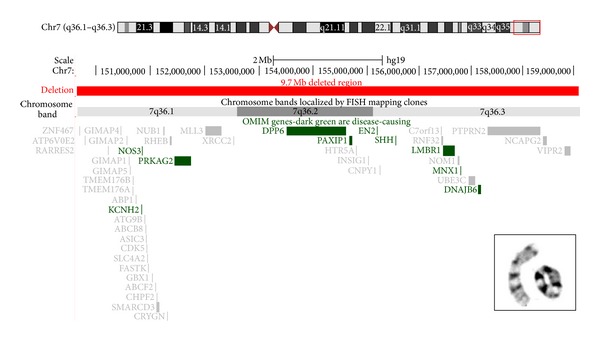
Cytogenetic and molecular analysis of ring chromosome 7. Partial karyotype showing the normal chromosome 7 and r(7) (p22.3q36.1) (inset). Ideogram illustrating the 9.7 Mb deleted region defined by aCGH (red bar) and UCSC Genome Browser display of OMIM genes within the deletion (GRCh37/hg19). Known disease-causing genes, including *SHH* and *MNX1*, are represented by darker bars.

Interphase fluorescence *in situ* hybridization (FISH) aneuploidy screening (AneuVysion Multicolor DNA Probe Kit, Abbott Molecular, Inc.) performed on uncultured villi was consistent with a female with two copies each of chromosomes X, 13, 18, and 21. Cytogenetic (G-band) analysis performed on cultured villi revealed a female karyotype with a chromosome 7 with the distal ends of the short (p) and long (q) arms fused at bands p22 and q36, respectively, resulting in a ring chromosome 7 [r(7)] (Figure 2, inset). In two of the twenty-one cells examined, the r(7) was larger in size, most likely the result of structural instability, a known behavior of ring chromosomes [1]. FISH using DNA probes for the subtelomere regions of 7p and 7q (probes pVYS230A (GenBank G31341) and pVYS231A (STS 2000H), respectively, from Abbott Molecular, Inc.) confirmed the deletion of both subtelomeric regions on the r(7). Parental karyotypes (G-band analysis of peripheral blood) were normal. The fetal karyotype was interpreted as 46,XX,r(7)(p22q36).ish r(7)(p22q36)(pVYS230A-, pVYS231A-)dn. The patient elected pregnancy termination by dilatation and curettage; therefore, an autopsy was not possible. 

Array comparative genomic hybridization (aCGH) was also performed on DNA isolated from cultured chorionic villi utilizing the 4x44K CytoChip ISCA oligonucleotide microarray platform (Agilent Technologies, Inc.). Analysis was performed with BlueFuse Multi 2.5 software (BlueGnome, Ltd.) with interpretation limited to chromosome 7. Array CGH confirmed the chromosome 7 subtelomere deletions and further refined the deletion breakpoints. A 7p copy number loss of 170 kb [arr 7p22.3(54,215-170,396x1)] and a 7q copy number loss of 9.7 Mb [arr 7q36.1q36.3(149,415,502-159,118,537x1)] were detected (GRCh37/hg19) and interpreted to be terminal. The 7p deleted region does not encompass any known genes while the 7q region contains 44 genes, namely, *SHH *and *MNX1* at 7q36.3 (Figure 2). Mutation or cytogenetic disruption of *SHH* (MIM 600725), which plays a significant role in early embryonic patterning, is known to cause HPE; thus, haploinsufficiency of *SHH* is likely contributing to the phenotype in this fetus. Additionally, loss of *MNX1* causes Currarino syndrome; however, features of this disorder (partial sacral agenesis, presacral mass, and anorectal malformation) were not apparent on ultrasound examination.

## 3. Discussion

Constitutional ring chromosomes occur in approximately 1/30–60,000 births and are inherited in no more than 1% of cases [1, 2]. Independent of the chromosome involved, a common “ring syndrome” phenotype has been described which includes extreme growth failure without major malformation, few or no minor congenital anomalies, and mild to moderate intellectual disability [1]. Molecular cytogenetic techniques are necessary to determine the extent of loss (if any) of genetic material at the ring chromosome breakpoints, and phenotypic severity has been shown to correlate with deletion size [1]. Ring chromosome 7 is a rare finding, with only 17 cases reported in the literature [3]. The most common clinical findings associated with r(7) include growth delay, microcephaly, and dermatologic abnormalities. 

To the best of our knowledge, ours is the first reported case of a prenatally identified r(7) that has been molecularly characterized. This finding was made subsequent to a first trimester diagnosis of HPE, a brain malformation in which the forebrain fails to completely separate into two hemispheres during development. HPE is a phenotypically variable disorder with heterogeneous etiology including both genetic and environmental causes. Numerical and structural chromosomal abnormalities account for up to 68% of prenatally detected HPE [4], while single gene defects (e.g., MIM 142945) and environmental factors underlie most of the remaining cases. The estimated prevalence of HPE in liveborn children is less than 1/10,000, but the incidence may be as high as 1/250 in first trimester conceptuses [5]. Only two cases of r(7) have been reported in infants with HPE [6, 7], although a subtelomeric deletion of 7q36 was confirmed by FISH in only one of these cases [6]. In our case, we confirmed subtelomeric deletions on both chromosome arms by FISH and further delineated the size, location, and gene content of the deleted regions through the use of aCGH. Importantly, aCGH demonstrated loss of *SHH*, consistent with the fetal diagnosis of HPE. While the use of cytogenetic microarrays for prenatal diagnosis is currently not standard practice, this case provides an example of its utility in characterizing a cytogenetic abnormality that has been associated with a spectrum of clinical features. As additional cases of molecularly characterized r(7) are reported in the literature, genotype-phenotype correlations may be facilitated in individuals diagnosed with this chromosome abnormality.
